# Cartilage 3D bioprinting for rhinoplasty using adipose‐derived stem cells as seed cells: Review and recent advances

**DOI:** 10.1111/cpr.13417

**Published:** 2023-02-12

**Authors:** Chong Zhang, Guanhuier Wang, Hongying Lin, Yujia Shang, Na Liu, Yonghuan Zhen, Yang An

**Affiliations:** ^1^ Department of Plastic Surgery Peking University Third Hospital Beijing China; ^2^ Key Laboratory of Structure‐Based Drug Design & Discovery of Ministry of Education, College of Traditional Chinese Materia Medica Shenyang Pharmaceutical University Shenyang China

## Abstract

Nasal deformities due to various causes affect the aesthetics and use of the nose, in which case rhinoplasty is necessary. However, the lack of cartilage for grafting has been a major problem and tissue engineering seems to be a promising solution. 3D bioprinting has become one of the most advanced tissue engineering methods. To construct ideal cartilage, bio‐ink, seed cells, growth factors and other methods to promote chondrogenesis should be considered and weighed carefully. With continuous progress in the field, bio‐ink choices are becoming increasingly abundant, from a single hydrogel to a combination of hydrogels with various characteristics, and more 3D bioprinting methods are also emerging. Adipose‐derived stem cells (ADSCs) have become one of the most popular seed cells in cartilage 3D bioprinting, owing to their abundance, excellent proliferative potential, minimal morbidity during harvest and lack of ethical considerations limitations. In addition, the co‐culture of ADSCs and chondrocytes is commonly used to achieve better chondrogenesis. To promote chondrogenic differentiation of ADSCs and construct ideal highly bionic tissue‐engineered cartilage, researchers have used a variety of methods, including adding appropriate growth factors, applying biomechanical stimuli and reducing oxygen tension. According to the process and sequence of cartilage 3D bioprinting, this review summarizes and discusses the selection of hydrogel and seed cells (centered on ADSCs), the design of printing, and methods for inducing the chondrogenesis of ADSCs.

## Graphical abstract

Cartilage 3D bioprinting for rhinoplasty using ADSCs as seed cells: When fabricating tissue‐engineered cartilage, the hydrogel‐based bio‐ink containing seed cells and additives is printed layer by layer to form the targeted construct, combined with other artificial materials or alone, hierarchical or homogeneous. ADSCs stand out from the candidates, especially those from thigh adipose tissue. When mixed with chondrocytes in appropriate proportions (1:3–3:1), more satisfactory cartilage regeneration results can be achieved. Besides, other methods of promoting the chondrogenesis of ADSCs are also imposed.

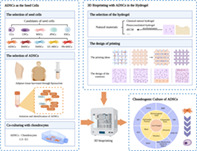

## 1 INTRODUCTION

Trauma, burn, tumour, surgery, or congenital malformation of nasal cartilage may compromise a nasal deformity or lead to nasal airway dysfunction, affecting the aesthetics and utility of the nose.^1^ Rhinoplasty usually requires trimming of nasal cartilage and implantation of grafts. Therefore, finding the most suitable and compatible cartilage graft material is essential. Ideally, the engineered cartilage graft should have properties to meet the clinical needs of the recipient site, including being able to withstand the external forces in the nasal reconstructive environment, biocompatible, and biologically active to support and promote tissue integration and healing. Besides, it should be readily available or easy to access without contributing to donor site morbidity. Moreover, from a surgical point of view, the graft material needs to be big enough in the case of intraoperative trimming to meet the needs of patients.

Currently, commonly used graft materials include autologous cartilage, allografts, and synthetic materials. Autologous cartilage, especially nasal septum cartilage, is an ideal cartilage source. However, the quantity of autologous cartilage is limited, let alone that harvesting auricular cartilage and costal cartilage will inevitably produce additional incisions, which could result in donor site complications. Widely sourced materials such as allografts and synthetic materials have been used as alternatives to autologous cartilage grafts. Nevertheless, allografts are more susceptible to resorption and infection, while the use of synthetic materials may lead to devastating complications. With further advances in tissue engineering technology, it will be possible in the future to address the issues related to the source of grafts in rhinoplasty by constructing ideal tissue‐engineered cartilage. Seed cells, scaffold materials, and growth factors were researched and improved to achieve optimization of engineered cartilage.^2^ Techniques of constructing scaffolds, including 3D bioprinting (such as inkjet bioprinting, laser‐assisted bioprinting, extrusion‐based bioprinting, acoustic bioprinting, stereolithography bioprinting, and magnetic bioprinting), direct moulding methods (such as cell sheet stacking, lithography, and injection moulding), and a group of methods for constructing porous scaffolds (such as electrospinning, phase separation, freeze‐drying, and self‐assembly), are also important to elevating cartilage performance.^3^, ^4^, ^5^, ^6^ Nowadays, 3D bioprinting, as a form of cell‐laden bottom‐up additive manufacturing, has become one of the most promising and advanced tissue engineering methods.

Adipose tissue is widely distributed in various parts of the human body. Adipose‐derived stem cells (ADSCs) are undifferentiated mesenchymal stem cells (MSCs) in adult adipose tissue with osteogenic, chondrogenic, and myogenic differentiation potential. The ADSCs play an important role in tissue engineering for advantages such as abundant sources and easy acquisition. In addition, exosomes secreted by ADSCs can promote cartilage regeneration, cell migration, and differentiation as a biological additive. For 3D bioprinting ideal highly bionic tissue‐engineered cartilage, it is crucial to successfully induce seed cells such as ADSCs to differentiate into chondrocytes (CCs) and secrete cartilage extracellular matrix (ECM). Researchers have attempted to achieve optimal cartilage by simulating the natural physicochemical environment in vivo, such as adding appropriate growth factors, applying biomechanical stimuli, and reducing oxygen tension.

**FIGURE 1:**
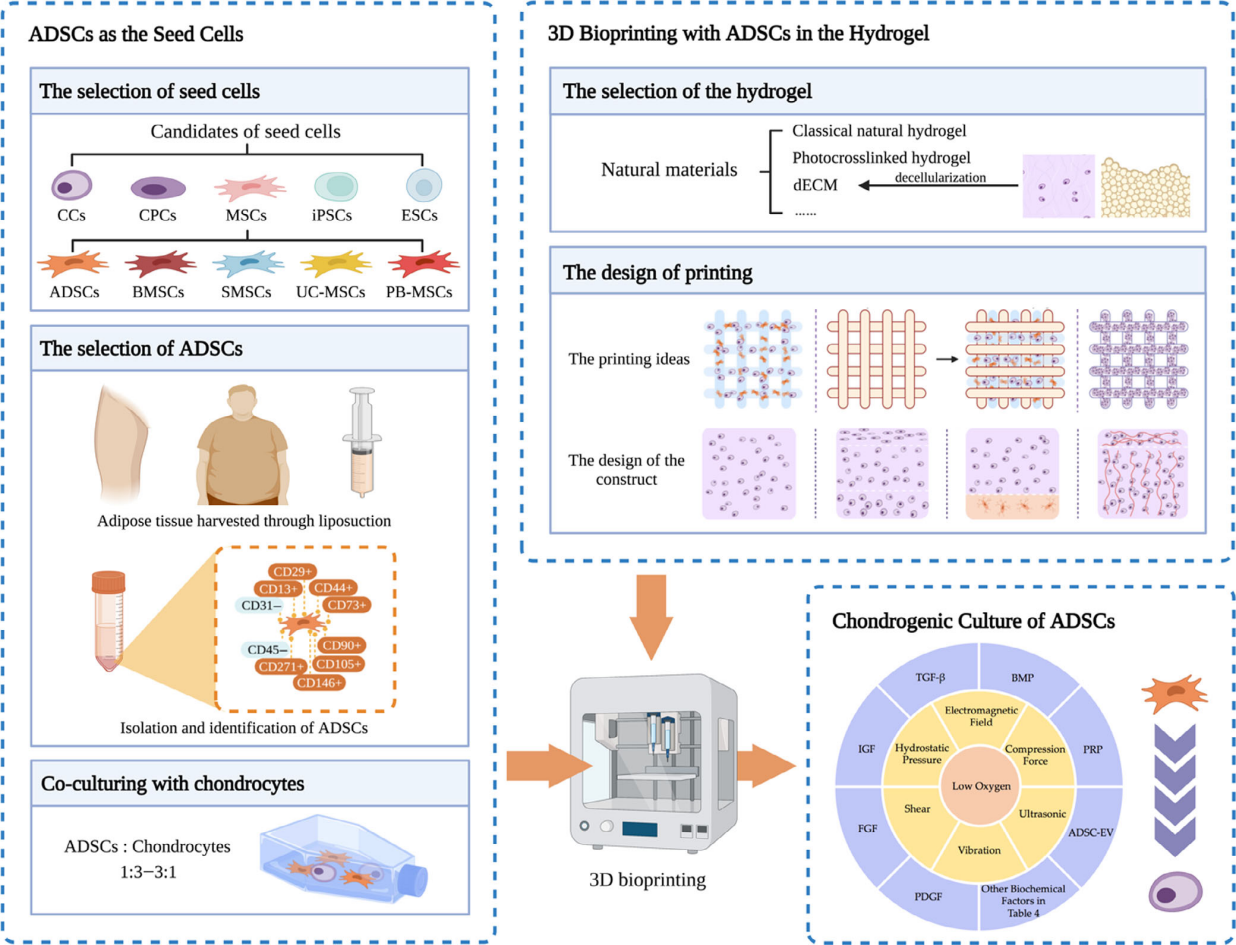
Cartilage 3D bioprinting for rhinoplasty using adipose‐derived stem cells (ADSCs) as seed cells: When fabricating tissue‐engineered cartilage, the hydrogel‐based bio‐ink containing seed cells and additives is printed layer by layer to form the targeted construct, combined with other artificial materials or alone, hierarchical or homogeneous. ADSCs stand out from the candidates, especially those from thigh adipose tissue. When mixed with chondrocytes in appropriate proportions (1:3–3:1), more satisfactory cartilage regeneration results can be achieved. Besides, other methods of promoting the chondrogenesis of ADSCs are also imposed. ADSC‐EV, ADSC‐derived extracellular vesicle; BMP, bone morphogenetic protein; BMSCs, bone marrow‐derived MSCs; CCs, chondrocytes; CPCs, chondroprogenitor cells; dECM, decellularized extracellular matrix; ESCs, embryonic stem cells; FGF, fibroblast growth factor; IGF, insulin‐like growth factor; iPSCs, induced pluripotent stem cells; MSCs, mesenchymal stem cells; PB‐MSCs, peripheral blood‐derived MSCs; PDGF, platelet‐derived growth factor; PRP, platelet‐rich plasma; SMSCs, synovium‐derived MSCs; TGF‐β, transforming growth factor β; UC‐MSCs, umbilical cord blood‐derived MSCs.

In this review, we summarize and discuss the selection of hydrogel and seed cells (centered on ADSCs), the design of printing, and the methods for inducing the chondrogenesis of ADSCs, according to the process and sequence of cartilage 3D bioprinting (Figure 1). We first introduce the current 3D bioprinting methods and reviewed the commonly used hydrogel scaffold materials and their progress. Then we mainly introduce the application of ADSCs in tissue engineering cartilage: It can be used as seed cells laden in bio‐ink and as the source of extracellular vesicles (EVs) to induce chondrogenesis of cells. Other methods of promoting chondrogenic differentiation of ADSCs were also described. Finally, we summarized and prospected the future directions of 3D bioprinting tissue‐engineered cartilage.

## 2 3D BIOPRINTING WITH ADSCs IN HYDROGELS

### 2.1 The hydrogel‐based 3D bioprinting

3D bioprinting is a form of bottom‐up additive manufacturing and tissue engineering technology. Before the printing process begins, an individualized model of the patient is constructed. With the help of medical imaging techniques, usually computerized tomography scan and magnetic resonance imaging, we can reconstruct the patient's nasal shape and use it as a basis to design the model according to the patient's needs, and then import the designed model into the 3D bioprinter. After that, bio‐ink containing seed cells, growth factors, and other additives is printed layer by layer to form the construct. And, after a specific in vitro culture, the personalized target tissue is finally constructed. 3D bioprinting technology can be roughly divided into three types: droplet, extrusion, and photocuring bioprinting, which are respectively suitable for bio‐ink materials with different curing mechanisms. According to the needs of experimental design, the material of bio‐ink is selected according to their biological, biophysical, and biochemical properties. The corresponding 3D bioprinting technique is then selected to construct the scaffold. Several excellent reviews have compared and discussed their advantages and disadvantages, which would not be repeated here.^7^, ^8^ Each step of 3D bioprinting will determine the quality of the final construct and the bio‐ink based on the hydrogel is the critical core. Hydrogels, a 3D network of molecules composed of hydrophilic polymer chains, can be designed into any shape, size, or form and absorb up to a thousand times their dry weight in a water‐rich environment.^9^, ^10^ With the development of 3D bioprinting technology, the hydrogel‐based system has become a prime candidate as the carrier for cells for a variety of tissue engineering applications.

The ideal bio‐ink should have good biocompatibility, biodegradability, and sufficient mechanical properties to support nasal morphology and mimic the natural ECM. In addition, the effect of the hydrogel material and its crosslinking process on the seed cells encapsulated needs to be considered. Since mechanical properties and biocompatibility are often challenging to achieve simultaneously, bio‐ink of a single formulation is rarely used at present, and mixed bio‐ink has gradually become the mainstream.

### 2.2 Hydrogels for cartilage tissue engineering and their general properties

**FIGURE 2:**
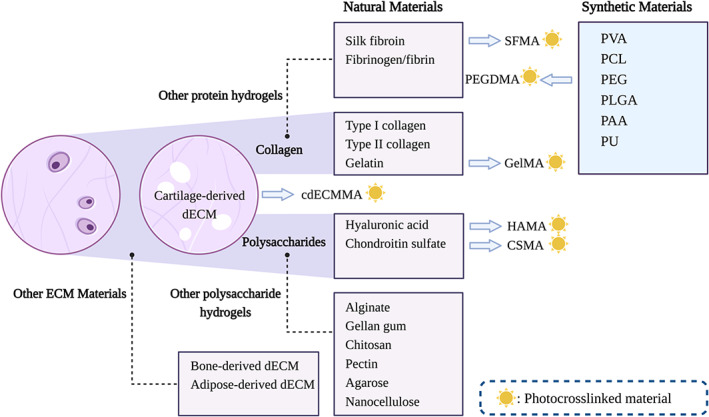
Natural and synthetic materials for cartilage 3D bioprinting: The natural materials for cartilage 3D bioprinting are mainly matrix components or proteins and polysaccharides with similar properties. Besides, some synthetic materials with high mechanical properties are also widely used. The methacrylated photocrosslinked materials are marked with a sign in the shape of the sun (yellow) on their right side. cdECMMA, methacrylated cartilage‐derived dECM; CSMA, methacrylated chondroitin sulfate; dECM, decellularized ECM; ECM, extracellular matrix; GelMA, methacrylated gelatin; HAMA, methacrylated hyaluronic acid; PAA, polyacrylic acid; PCL, polycaprolactone; PEG, polyethylene glycol; PEGDMA, polyethylene glycol dimethacrylate; PLGA, polylactic acid‐glycolic acid copolymer; PU, polyurethane; PVA, polyvinyl alcohol; SFMA, methacrylated silk fibroin.

The materials of bio‐ink for cartilage 3D bioprinting can be divided into natural and synthetic ones (Figure 2). The natural materials are mainly cartilage matrix components such as hyaluronic acid (HA), collagen, chondroitin sulphate (CS), decellularized ECM (dECM), and so forth. Besides, other proteins and polysaccharides with similar properties are also widely used, such as silk fibroin (SF), agarose, gellan, gelatin, alginate, chitosan, and pectin. While synthetic materials mainly include polycaprolactone (PCL), polylactic acid‐glycolic acid copolymer, polyurethane, polylactic acid, polyacrylic acid (PAA), and polyethylene glycol (PEG). Bio‐ink is often printed in liquid or gel form and later cured. To balance the printability, mechanical properties, and biocompatibility of the bio‐ink, it is essential to have the proper forming and curing method. In general, the method is often determined by the properties of the bio‐ink itself.

The common crosslinking mechanisms of hydrogels include physical crosslinking, chemical crosslinking, and enzymatic crosslinking. Physical crosslinking allows effective forming without any exogenous factors and minimizes chemical toxicity. Physical entanglement of the polymer chains occurs during the gelation of thermally driven hydrogels. This happens with natural polymers like gelatin and synthetic polymers like PAA.^11^ Molecular self‐assembly is a commonly used strategy for protein‐based hydrogels such as collagen through weak noncovalent bonds like ionic bonds, hydrogen bonds, hydrophobic interactions, and van der Waals force.^12^ In addition, physical crosslinking can also be based on chelation or electrostatic interaction.^11^ Natural ionic polysaccharides such as alginate and pectin can gel in the presence of divalent cations such as calcium ions.^13^ Chemical crosslinking is based on covalent bonding between polymer chains, including condensation reactions, radical polymerization, and aldehyde complementation.^10^ When compared with physical crosslinking, the covalent bonding chemical crosslinking is more stable and tunable, substantially improving the spatiotemporal precision and flexibility during the printing‐curing process.^11^ Photo‐initiator (PI) is often added to modify the functional groups of free radical polymerization to obtain photocrosslinking properties of hydrogels. Photocrosslinked hydrogels, such as methacrylated gelatin (GelMA) and PEG dimethacrylate (PEGDMA), with crosslinking rates and degrees easy to control, are widely used in 3D bioprinting of cartilage tissue. Finally, enzymatic crosslinking is commonly used to form fibrin (thrombin) and gelatin (glutamic‐aminotransferase).

Cartilage tissue consists mainly of CCs and ECM, which consist of proteoglycans and collagen fibrils (type II) to form a solid structure. In addition, more CS is observed in the matrix around the cartilage lacunae, which is called the cartilage capsule. The classic natural biological bio‐inks are selected and combined from the components of the cartilage ECM. In addition, the dECM is one of the most promising materials for cell encapsulation. Besides, many other biocompatible and structurally similar protein and polysaccharide materials are used. Natural hydrogels can become photocurable by methacrylate. GelMA and other materials made on this basis have become the most popular materials in 3D bioprinting.

The hydrogel materials currently used for cartilage 3D bioprinting are only a fraction of the available hydrogels for tissue engineering. Not to mention that there are still many possibilities for developing tissue engineering hydrogels. The composition of natural cartilage tissue often inspires hydrogel formulations, and the cartilage matrix components are complex and diverse. Research related to decellularized matrix also provides researchers with another different line of thought to explore the natural combinations.

### 2.3 The emerging bio‐ink formulations and the shift in printing ideas

#### 2.3.1 The hybrid hydrogel formulations for cartilage tissue engineering

Nowadays, natural hydrogels are mainly used for the 3D bioprinting of cartilage tissue. Natural hydrogels commonly have good biocompatibility, and their mechanical properties are lower than that of natural cartilage. Synthetic materials, however, can provide high mechanical strength. Therefore, in the preparation of bio‐ink, a mixture is often used to complement their strengths and weaknesses, or other additives are added to natural bio‐inks to improve their mechanical properties. Researchers have continuously explored and modified hydrogels and improved bio‐ink formulations in recent years. Current studies in the field mainly focus on mixtures of bio‐ink with “good biocompatibility” (such as dECM, HA) and “photocrosslinking properties” (such as, methacrylated hyaluronic acid [HAMA], GelMA, PEGDMA) or “good mechanical properties “(such as silk protein, synthetic materials; Table 1).

**TABLE 1:** Summary of cartilage 3D bioprinting studies in recent years, in which various hybrid hydrogel formulations and seed cells were used.

Seed cells	The cell‐laden hydrogels	Sparkles	Reference
Articular CCs	GelMA, PEGDMA, and gelatin	Integrated cartilage‐mimetic tissue with a biosensing platform, and developed an odour‐perceptive nose‐like hybrid	^14^
	HAMA, pHPMA‐lac‐PEG, and PCL	Limited fibrocartilage formation, generated complex 3D constructs with mechanical stiffness	^15^
	GelMA and gellan gum	Increased the construct stiffness, supported chondrogenesis	^16^
	pHPMA‐lac‐PEG, CSMA, or HAMA	Increased the storage modulus of polymer mixtures, decreased the degradation kinetics in crosslinked hydrogels	^17^
	GelMA and HAMA	Opened up the perspective of hybrid and zonal stratification bio‐ink to generate more complex cartilage structures	^18^
	Alginate and sub‐micron PLA	Higher Young's modulus and cell viability	^19^
	HA and alginate or PLA	Good printability, degradation, mechanical properties, retained more than 85% cell viability	^20^
	GelMA, HAMA, and CSMA	Enhanced cartilage formation, promoted matrix distribution, improved mechanical properties	^21^
Auricular chondrocytes	PAOXA, alginate, and NFC	Broader tuning potential of mechanical properties increased cytocompatibility	^22^
	cdECMMA	Printed in ear shape, adequate mechanical properties and structural integrity	^23^
NSCs	NFC and alginate	Increased shape fidelity, printing resolution, and storage modulus correlating	^24^
	Gelatin and alginate‐di‐aldehyde	Good cell viability, increased expression of cartilage‐specific markers, not degrading within 28 days	^25^
BMSCs	SF and dECM	Good mechanical strength, released chondrogenic growth factors, enhanced chondrogenesis of BMSCs	^26^
	Alginate‐GelMA interpenetrating network and alginate sulfate	Improved mechanical properties, continuously released TGF‐β3 with high‐affinity binding, promoting chondrogenesis and suppressing hypertrophy of BMSCs	^27^
	Acrylated peptides (RGD) and PEGDMA	Enhanced the osteogenic and chondrogenic differentiation of BMSCs with minimal printhead clogging	^28^
	PEGDMA and GelMA	Bionic compressive modulus and promoted differentiation	^29^
	Alginate, GelMA, CS, amino ethyl methacrylate, and HAMA	Favoured the differentiation of BMSCs toward hypertrophic cartilage, supported the hypothesis that too crosslinked matrix does not allow for proper macromolecular diffusivity and hinders cartilage development	^30^
	Fibrin‐HA and HA‐Tyramine	Supported cell migration and enhanced cartilage regeneration	^31^
	Alginate, GelMA, and β‐tricalcium phosphate	Regulated the differentiation of BMSCs to form osteochondral tissue calcification zone	^32^
ADSCs	Hydroxybutyl chitosan and oxidized CS	Good injectability and controllable shape	^33^
iPSCs	Gelatin and PU	Convenient printing processes, tunable mechanical properties, and degradation rates	^34^
ATDC5	PVA‐based materials and solubilized cdECM	Balanced compression modulus, enhanced cell viability, and modulated the swelling ratio of hydrogel	^35^

Abbreviations: ADSCs, adipose‐derived stem cells; ATDC5, a teratocarcinoma‐derived chondrogenic cell line; BMSCs, bone marrow‐derived mesenchymal stem cells; CCs, chondrocytes; cdECM, cartilage‐derived decellularized extracellular matrix; cdECMMA, methacrylated cartilage‐derived decellularized extracellular matrix; CS, chondroitin sulfate; CSMA, methacrylated chondroitin sulfate; dECM, decellularized extracellular matrix; GelMA, methacrylated gelatin; HA, hyaluronic acid; HAMA, methacrylated hyaluronic acid; iPSCs, pluripotent stem cells; NSCs, nasoseptal chondrocytes; PAOXA, poly(2‐alkyl‐2‐oxazoline); PCL, polycaprolactone; PEGDMA, polyethylene glycol dimethacrylate; pHPMA‐lac‐PEG, polyethylene glycol mid‐block flanked by two poly(N‐(2‐hydroxypropyl) methacrylamide mono/dilactate) outer blocks; PLA, polylactic acid; PU, polyurethane; PVA, polyvinyl alcohol; SF, silk fibroin; TGF‐β3, transforming growth factor β3.

#### 2.3.2 The shift in 3D bioprinting ideas

**FIGURE 3:**
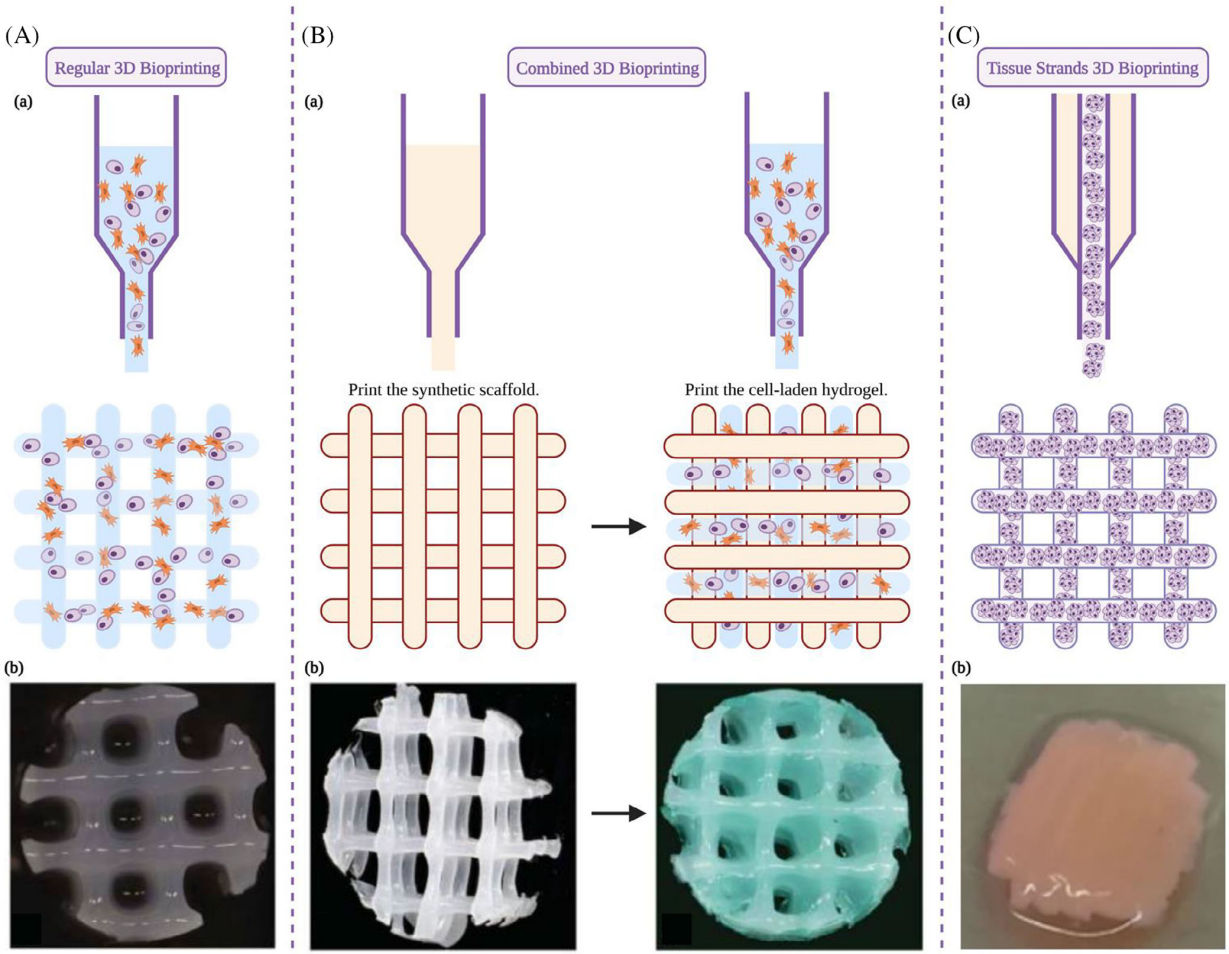
Diversified 3D bioprinting ideas: (A) Regular 3D bioprinting: (a) Schematic diagram; (b) Top view of a 3D bioprinted hydrogel construct.^36^ (B) Combined 3D bioprinting: (a) Schematic diagram; (b) Top view of a 3D printed synthetic scaffold and the composite construct after the 3D bioprinting of cell‐laden hydrogel.^36^ (C) Tissue strands 3D bioprinting: (a) Schematic diagram; (b) Top view of a 3D bioprinted construct formed by printed tissue strands.^37^

As bioprinting materials have continued to progress over the years, some researchers have shifted their attention from traditional 3D bioprinting to more diverse hybrid printing. In some studies, 3D scaffolds with excellent mechanical properties are printed with synthetic materials and filled with cell‐laden hydrogels later (Figure 3). The combination of two tissue‐engineered cartilage methods provides another direction to optimize engineered cartilage. In this way, synthetic materials represented by PCL have played a more critical role in 3D bioprinting with their outstanding mechanical properties (Table 2). Besides, Wu et al.^37^ used pre‐differentiated ADSCs to form cartilage scaffold‐free tissue strands and constructed zonally stratified cartilage, in which collagen fibres are aligned with designated orientation in each zone imitating the anatomical regions and matrix orientation of native articular cartilage and with satisfying compression modulus at the same time.

**TABLE 2:** Studies combining cell‐loaded hydrogels with scaffolds.

Seed cells	The cell‐laden hydrogel	The scaffold material	Reference
Pig ADSCs	Collagen type I and HA	PCL	^38^
Pig auricular CCs			
Pig tracheal CCs			
Rabbit auricular CCs	GelMA	PLA	^39^
Goat auricular CCs	Alginate	PCL	^40^
Horse articular CCs	Gelatin and dECM	PCL	^41^
Human ADSCs	GelMA and HA	PCL	^42^
Rabbit auricular CCs			
Human ADSCs	Alginate and dECM	PCL	^43^

Abbreviations: ADSCs, adipose‐derived stem cells; CCs, chondrocytes; dECM, decellularized extracellular matrix; GelMA, methacrylated gelatin; HA, hyaluronic acid; PCL, polycaprolactone; PLA, polylactic acid.

#### 2.3.3 The shift in the designs for 3D bioprinting constructs

**FIGURE 4:**
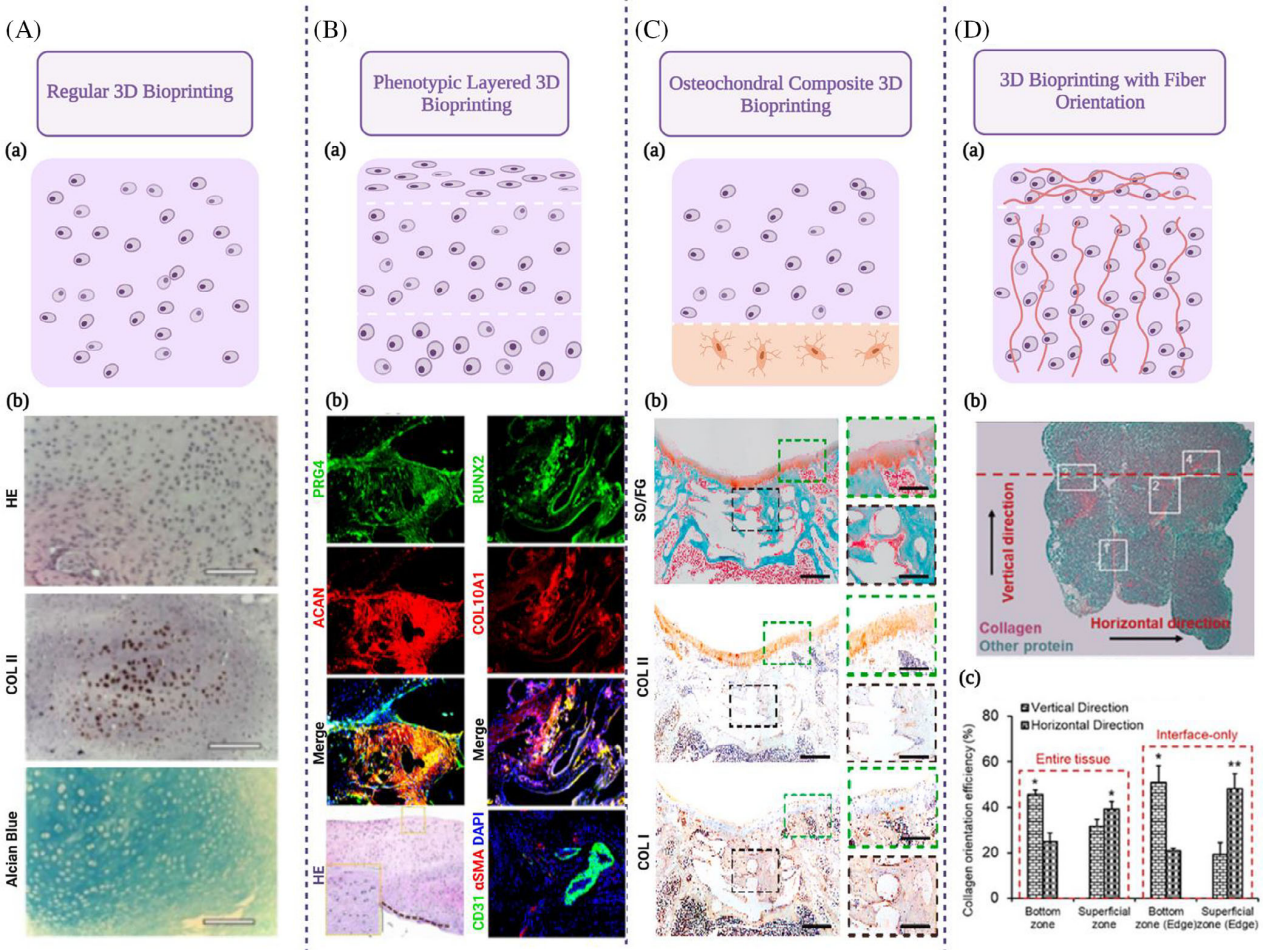
Diversified design in constructs: (A) Regular 3D bioprinting: (a) Schematic diagram; (b) Histological analysis of the cartilage tissue in animals (scale bar = 100 μm).^44^ (B) Phenotypic layered 3D bioprinting: (a) Schematic diagram; (b) The expression of PRG4 and ACAN in the superficial layer and ossification markers (RUNX2 and COL10A1) and microvessel ingrowth markers (CD31) in the deep layers.^45^ (C) Osteochondral composite 3D bioprinting: (a) Schematic diagram; (b) Histological analysis of the regenerated osteochondral tissue (scale bar 1 mm). The repair boundary of the cartilage surface (green dashed box) and the regenerated tissue of the subchondral bone layer (black dashed box) were respectively amplified (scale bar = 500 μm).^36^ (D) 3D bioprinting with fibre orientation: (a) Schematic diagram; (b) Sagittal sections of bioprinted cartilage with Picrosirius Red/Fast Green straining demonstrating the stratified arrangement and collagen organization in whole cartilage (scale bar = 500 μm); (c) Quantitative measurement of collagen alignment efficiency at the superficial and bottom zones of the entire tissue, as well as at the interfaces of tissue strands (*n* = 3, *p < 0.05 and **p < 0.01).^37^

In addition to the diversification in the modalities of 3D bioprinting, the requirements for the constructs are also changing (Figure 4). Conventional 3D bioprinting constructs are often homogeneous cartilage‐like tissue. However, the layered structure of cartilage tissue plays an important role in its physiology. Histologically, the nasal cartilage is divided into the superficial zone and the deep zone, composing a sandwich‐like three‐layer structure. When coming close to the deeper zone, cells gradually become larger, more rounded, and less frequent.^46^ Besides, the oxygen tension and nutrient availability of the tissue and the CCs population with hypertrophic and ossification markers such as Runt‐related transcription factor 2 (RUNX2) and collagen type X (COL X) are increasing.^47^ Therefore, the construction of hierarchical cartilage tissue with anisotropic properties is beneficial to achieving better results. Regrettably, most of the current studies that have carried out a hierarchical design have targeted articular cartilage for construction, and anisotropic tissue‐engineered nasal cartilage has yet to be investigated. With the perichondrium on its surface and a sandwich‐like structure rather than transiting into the subchondral bone, the nasal cartilage is different from the articular cartilage. However, nasal cartilage and articular cartilage are both hyaline cartilage, and the superficial and the uncalcified deep zones of the articular cartilage tissue are comparable to those of the nasal cartilage tissue,^48^ so the design idea of the layered tissue‐engineered articular cartilage in these studies can still be taken into reference when designing tissue‐engineered nasal cartilage.

In the study of Sun et al.,^45^ anisotropic cartilage constructs were generated by sequentially printing protein‐releasing and MSC‐laden hydrogels, and the ECM composition of different layers shared many characteristics of native cartilage, including the gradient expression of collagen type II (COL II), superficial localization of proteoglycan 4 (PRG4), and abundant presence of COL X in the deep zone, indicative of regenerated superficial zone articular cartilage and deep zone hypertrophic cartilage in the constructs. Considering the complicated requirements in osteochondral reconstruction, some studies have also constructed integrated osteochondral tissue by layered printing. In the study of Qiao et al.,^36^ a tri‐layered stratified scaffold was designed and fabricated, including a superficial cartilage layer with a lubricating and wear‐resistant surface, a deep cartilage layer, and a subchondral bone layer. Wu et al.^49^ fabricated a bilayer silk scaffold with a cartilage layer resembling native cartilage in surface morphology and mechanical strength and a porous subchondral bone layer loading bone morphogenetic protein (BMP)‐2 to facilitate the osteogenic differentiation of bone marrow‐derived MSCs (BMSCs). Liu et al.^50^ developed a biomimetic biphasic osteochondral scaffold with the layer‐specific release of stem cell differentiation inducers, in which the cartilage regeneration layer and the bone‐regeneration layer continue releasing kartogenin (KGN) and alendronate (ALN), respectively. Using the concept of constructing an osteochondral composite tissue, a promising dorsal nasal graft with gradient mechanical properties corresponding to nasal bone, nasal cartilage, and nasal tip can be constructed for rhinoplasty, achieving more satisfying postoperative effect. Finally, there are efforts to build biologically functional nasal cartilage. Jodat et al.^14^ proposed a proof‐of‐concept for an odour‐perceptive nose‐like hybrid, which is composed of a mechanically robust cartilage‐like construct and a biocompatible biosensing platform. Combining engineered cartilage‐like tissue with an electrochemical biosensing system, the hybrid construct could bring functional olfaction to specific airway disease biomarkers, explosives, and toxins, laying the foundation for functional bionic interfaces and humanoid robots. On the whole, with the increasing demand for bionicity on morphology and function, integrated printing of multilayered, composite tissue using diverse hydrogels is a promising direction for cartilage 3D bioprinting.

## 3 ADSCs AS THE SEED CELLS

**FIGURE 5:**
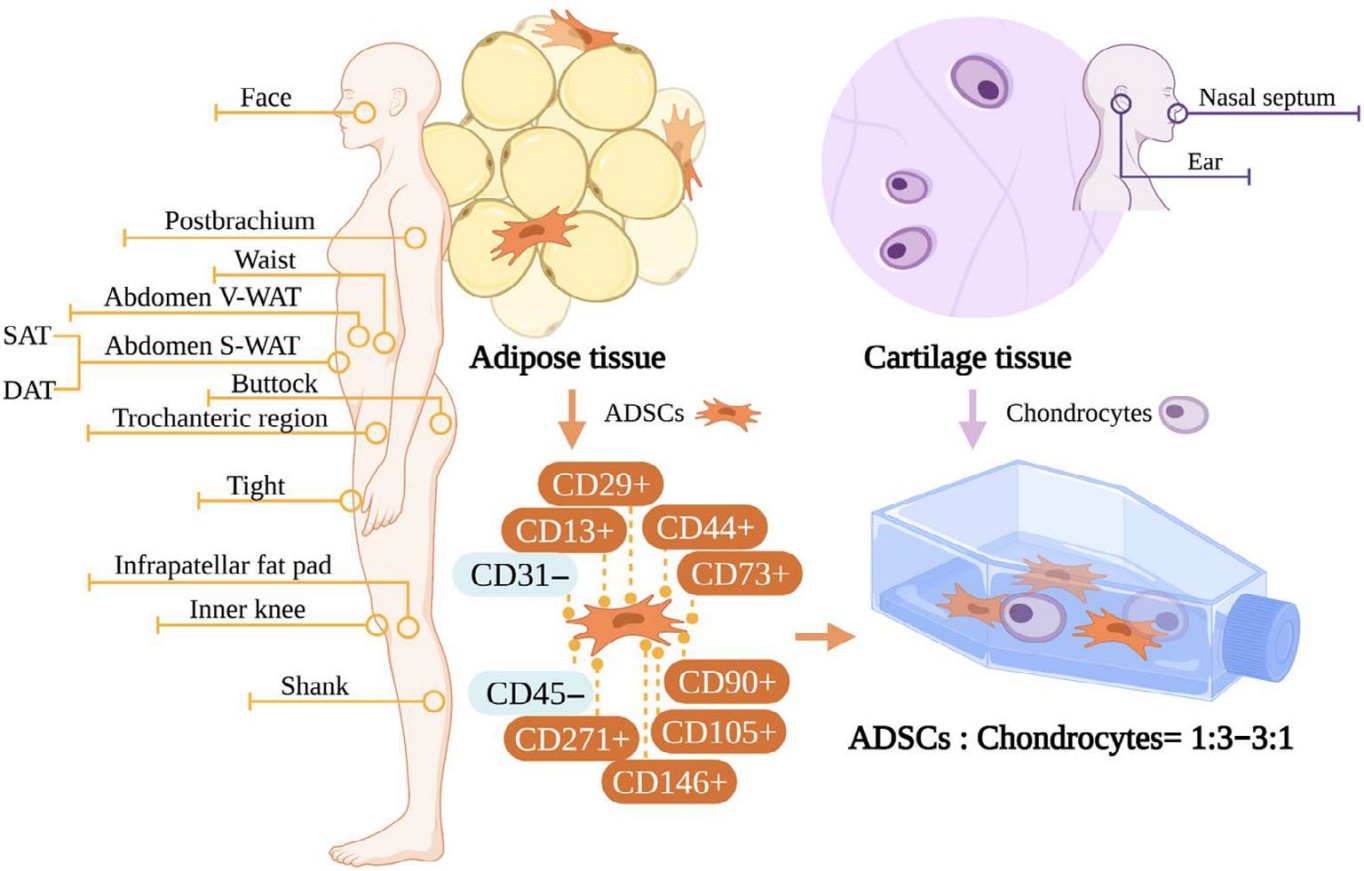
Adipose‐derived stem cells (ADSCs) are one of the most popular seed cells in cartilage tissue engineering. ADSCs with specific immunophenotypes can be harvested from adipose tissue from all parts of the body. When cocultured with chondrocytes from the nasal septum or ear, the ratio of ADSCs to chondrocytes is always from 1:3 to 3:1. DAT, deep layer adipose tissue; SAT, superficial adipose tissue; S‐WAT, subcutaneous white adipose tissue; V‐WAT, visceral white adipose tissue.

Seed cells are the basis of tissue engineering and an integral part of 3D bioprinting. In cartilage tissue engineering, the formation of the cartilage matrix depends on the seed cells encapsulated in the hydrogel. ADSCs are one of the most popular seed cells in cartilage tissue engineering, and where to obtain them, what phenotype to obtain, and how to culture them are all important topics (Figure 5).

### 3.1 The selection of seed cells

There are various seed cell options for the tissue engineering of nasal cartilage: CCs, chondroprogenitor cells (CPCs), MSCs, induced pluripotent stem cells, and embryonic stem cells.^51^ MSCs mainly include ADSCs, BMSCs, synovium‐derived MSCs (SMSCs), umbilical cord blood‐derived MSCs, and peripheral blood‐derived MSCs. Adipose tissue has a significantly higher concentration of MSCs than other sources, including the bone marrow, the dermis, the umbilical cord, the dental pulp, and the placenta.^52^ Nasal CCs and BMSCs used to be the most commonly used choice. While in recent years, the ADSCs have emerged as powerful competitors for their accessibility. In addition, the nasal CPCs also take their place in nasal cartilage tissue engineering.

ADSCs have become one of the most popular seed cells in cartilage 3D bioprinting, owing to their abundance, excellent proliferative potential, minimal morbidity during harvest, and lack of ethical considerations limitations.^53^ In the study of Mohamed‐Ahmed et al.,^54^ the differentiation ability and tissue specificity of BMSCs and ADSCs were different: BMSCs were superior to ADSCs in terms of osteogenic and chondrogenic differentiation, while ADSCs had more significant proliferation and adipogenic potential. However, MSCs are denser in adipose tissue than in bone marrow. In skeletally mature adults, the frequency of BMSCs in the bone marrow is between 0.001% and 0.002%, equating to several hundred BMSCs per millilitre of bone marrow. In contrast, ADSCs constitute about 1% of stromal vascular fraction (SVF) cells in digested adipose tissue, 500–1000 times more than that in marrow.^55^ Overall, ADSCs offer practical advantages as seed cells for cartilage regeneration.

### 3.2 The selection of ADSCs

#### 3.2.1 Where to access the adipose tissue?

In plastic surgery, if the patient also plans to undergo liposuction, ADSCs can be extracted directly from the adipose tissue obtained during the surgery. If not, it is necessary to find the most suitable site to harvest ADSCs.

Adipose tissue can be classified by morphology into white, brown, and beige subsets.^56^ White adipose tissue (WAT) distributes throughout the body, and different WAT depots have differences in cell composition and function. In human WAT, the subcutaneous WAT (S‐WAT) expands to store excess lipids, thereby preventing ectopic deposition of lipids and organ damage, while the main function of visceral WAT (V‐WAT) is to buffer and protect visceral organs.^57^ The abdominal V‐WAT depots are mainly located around the omentum, intestines, and perirenal areas. S‐WAT depots are distributed in the abdomen, buttock, thigh, face, other parts of the extremities, and so forth. In previous studies, S‐WAT was the primary source of adipose stem cells from the upper and lower abdomen, trochanteric region, inner thigh, knee, and flank.^58^ As early as 2008, Padoin et al.^58^ found a significant difference in the obtained cell concentration of different harvest sites: the concentration of the ADSCs in the lower abdomen and the inner thigh was higher than in other S‐WAT areas. Besides, Tsekouras et al.^59^ reported a significantly higher yield of ADSCs and SVFs from adipose tissue of the inner as well as the outer thigh when compared with those of the abdominal, waist, and inner knee regions. Subcutaneous adipose tissue in the abdominal region features a complex architecture: the superficial fascia separates superficial adipose tissue (SAT) from deep layer adipose tissue (DAT). The comparative study of Di Taranto et al.^60^ implied that SAT seemed to be a better source of ADSCs because SAT contained a higher stromal tissue compound, and the cells isolated from SAT displayed increased multipotency and stemness features, compared with DAT from the same harvesting site. In the study of Tang et al.,^55^ human S‐WAT ADSCs showed better chondrogenic potential and immunosuppression than V‐WAT ADSCs (V‐ADSCs) in vitro, although the proliferation of V‐ADSCs was significantly greater. Hendawy et al.^61^ analysed the ADSCs from different visceral adipose tissue depots of rats, suggesting that the mesenteric ADSCs showed a better chondrogenic differentiation potential than peri‐ovarian and peri‐renal ADSCs.

Although lipoaspirate from abdominal adipose tissue is the most popular and abundant source of ADSCs for cartilage repair, the infrapatellar fat pad (IFP) also offers a clinical source of ADSCs. ADSCs derived from IFP demonstrate higher chondrogenic capacity than those from S‐WAT, even the abdomen, which seemed to be the most appropriate place to get S‐WAT.^62^ When compared with ADSCs from knee S‐WAT, the IFP ADSCs showed greater amounts of glycosaminoglycans, superior expression of aggrecan (ACAN), SRY‐related HMG box 9 (SOX9), cartilage oligomeric matrix protein (COMP), and COL2A1, and lower expression of COL10A1 and COL1A1, and alkaline phosphatase release.^63^

In conclusion, for the general population of patients with nasal deformities, the thighs are the best donor sites of adipose tissue according to current studies, taking into account the difficulty of the procedure and the efficiency of ADSCs harvest. Abdominal S‐WAT is also a better choice considering the liposuction needs of different patients. In addition, IFP is also a good choice if the patient's need is for articular cartilage tissue when suffering from articular cartilage injury.

#### 3.2.2 What kind of ADSC is better?

From a broad perspective, human ADSCs express the MSC markers CD9, CD10, CD13, CD29, CD44, CD49d, CD49e, CD54, CD55, CD73, CD90, CD105, CD106, CD146, CD166, and stromal precursor antigen‐1 (STRO‐1), but they are negative for the haematopoietic cell lineage markers CD14, CD19, CD34, CD45, CD16, CD56, CD61, CD62E, CD104, and CD106, and are also negative for the endothelial cell markers CD31 and CD144.^64^ Flow cytometry is often used to screen cell surface immune markers. CD73+, CD90+, CD105+, CD11b− or CD14−, CD19− or CD79α−, CD34−, CD45−, and HLA‐DR− are considered to be classic surface markers of MSCs.^65^ However, there is no unified screening standard for ADSCs, and disagreements remain over markers like CD34.^66^ The main surface markers with the significance of identification include CD13+, CD29+, CD44+, CD73+, CD90+, CD105+, CD31−, and CD45−.^66^, ^67^

ADSCs from different body parts have different phenotypes, while cellular heterogeneity exists even among the ADSCs isolated from the same adipose depot. Besides, the phenotypes of ADSCs evolve with expansion in vitro, and some studies have been conducted on the temporal changes of ADSC surface markers during expansion in vitro.^68^, ^69^ Cultured ADSCs from the same source can be further sorted by cell surface immunophenotype into subpopulations with a distinct differentiation potential into the cell type of interest.^57^ CD105 has been known as a relatively specific marker for identifying MSCs. In the study of Jiang et al.,^70^ CD105+ cells exhibited a much stronger chondrogenic potential than CD105‐ cells, with higher gene expression of COL II and ACAN and could form a homogeneous cartilage‐like tissue in vitro. CD271 is a marker of BMSCs with enhanced differentiation capacity for bone or cartilage repair. In the study of Kohli et al.,^71^ the selective isolation of CD271+ ADSCs resulted in less angiogenesis and better cartilage repair of osteochondral tissue damage, suggesting that the CD271+ ADSCs were more conducive to the repair of mature vascularless tissue like cartilage. CD146 is a transmembrane glycoprotein and an immunoglobulin superfamily adhesion molecule. CD146 was initially known as an endothelial biomarker and recognized as a marker for pericytes, which have been identified as the natural ancestors of MSCs. In the study of Li et al.,^72^ the CD146+ ADSCs showed a high expression of stem cell and pericyte markers, good viability, immune characteristics to avoid allogeneic rejection, and the potential to promote better cartilage repair. Finding the fewest and most precise phenotypic combinations helps to screen out ADSCs that are most suitable for cartilage tissue engineering in the future. Research on the immunophenotypes that support the chondrogenesis of ADSCs is still limited, while the previously mentioned studies on CD105, CD146, and CD271 provided us with hints to some extent.

In addition to sorting ADSCs based on immunophenotype, methods that can mechanically sort cells based on their physical properties have been proposed. Gonzalez‐Cruz et al.^73^, ^74^ demonstrated that an immunolabel‐free approach could be employed to sort ADSCs into subpopulations with a variable propensity to differentiate into adipocytes, CCs, and osteoblasts, based on the elastic and viscoelastic properties of ADSCs, and the chondrogenesis of ADSCs was positively correlated with elastic modulus and apparent viscosity. Although few studies have been done, the mechanical sorting method is more direct and easier to use than the immunophenotypic sorting method, which broadens the methods of sorting ADSCs.

### 3.3 A mix of seed cells: co‐culturing with the chondrocytes

Despite all the advantages of ADSCs as seed cells mentioned earlier, the application of MSCs alone as seed cells could result in unstable and hypertrophic cartilage tissue with a high tendency to ossification.^75^ Meanwhile, the application of CCs alone as seed cells shows obvious limitations since they have limited proliferation ability and are difficult to harvest in quantities.

Hence, researchers also devised a scheme for mixing seed cells in addition to a mixture of materials. BMSCs have a synergistic effect with articular CCs when co‐cultured. Similarly, this effect is also present with ADSCs and CCs.^62^ Culturing ADSCs mixed with CCs can promote cartilage tissue formation.^76^ Signals from CCs could enhance the expression of chondrogenic markers such as COL II, ACAN, SOX9, or glycosaminoglycan (GAG) and effectively promote chondrogenic differentiation of ADSCs, by direct interaction or in the form of soluble factors, including transforming growth factor β (TGF‐β), insulin‐like growth factor (IGF)‐1, BMP‐2, fibroblast growth factor (FGF)‐2, and cytokine‐like 1.^77^, ^78^ Besides, ADSCs can differentiate directly into CCs on the one hand, and release paracrine factors to promote CCs growth on the other hand.^79^ In terms of effect, co‐culture induced the same or higher levels of expression of chondrogenic markers compared with growth factors induction. Moreover, the two may act in synergy work.^77^ Many researchers have widely used the multi‐seeded cell scheme, while some of them used BMSCs instead of ADSCs (Table 3).

**TABLE 3:** Studies on 3D bioprinting of cartilage with co‐culturing MSCs and CCs.

Seed cells	The cell‐laden hydrogels	Recommended scheme	Reference
Human BMSCs	Aminoethyl CSMA, GelMA, HAMA, and alginate	BMSCs: CCs = 3:1	^80^
Human articular CCs			
Human BMSCs	NFC	BMSCs: chondrocytes = 4:1	^81^
Human nasal CCs			
Pig ADSCs	Type I collagen and HA	ADSCs: CCs = 5:1 (in 1:1/ 2:1/ 5:1/ 10:1)	^38^
Pig auricular and tracheal CCs			
Human ADSCs	Adipose‐derived dECM	ADSCs: CCs = 9:1 (in 5:1/ 9:1)	^79^
Pig auricular CCs			
Human ADSCs	Type I collagen and HA	ADSCs: CCs = 1:3 (in 1:0/ 3:1/ 1:1/ 1:3/ 0:1)	^75^
Human articular CCs			
Human ADSCs/BMSCs	Alginate	BMSCs: CCs = 4:1 (in BMSCs/ ADSCs: CCs = 4:1)	^82^
Bovine articular chondrocytes			
Human BMSCs	GelMA and HAMA	BMSCs: CCs = 3:1/ 1:1 (no difference; in 1:0/ 3:1/ 1:1/ 0:1)	^83^
Human NSPCs			
Pig BMSCs	Alginate‐GelMA and PCL	BMSCs: CCs = 3:1	^84^
Pig chondrocytes			
Human ADSCs	Alginate and PCL	ADSCs: CCs = 1:1	^40^
Rabbit articular CCs			
Human BMSCs	Alginate	BMSCs: CCs = 2:1 (in 1:0/ 2:1/ 1:1/ 1:2/ 0:1)	^85^
Rabbit articular CCs			
Human articular CCs			

Abbreviations: ADSCs, adipose‐derived stem cells; BMSCs, bone marrow‐derived mesenchymal stem cells; CCs, chondrocytes; CSMA, methacrylated chondroitin sulfate; dECM, decellularized extracellular matrix; GelMA, methacrylated gelatin; HA, hyaluronic acid; HAMA, methacrylated hyaluronic acid; NFC, nanofibrillated cellulose; NSPCs, nasoseptal primary CCs; PCL, polycaprolactone.

Taken together, there is no co‐identification in the ratio of ADSCs to CCs. Nevertheless, what is clear is that the induction effect of CCs is dose‐dependent: the higher the proportion of CCs, the more GAG was produced.^86^ On balance, a ratio from 1:3 to 3:1 of ADSCs to CCs may be a relatively practical and conservative choice (Figure 5). However, the most precise ratio needs to be further investigated.

## 4 CHONDROGENIC CULTURE OF ADSCs

**FIGURE 6:**
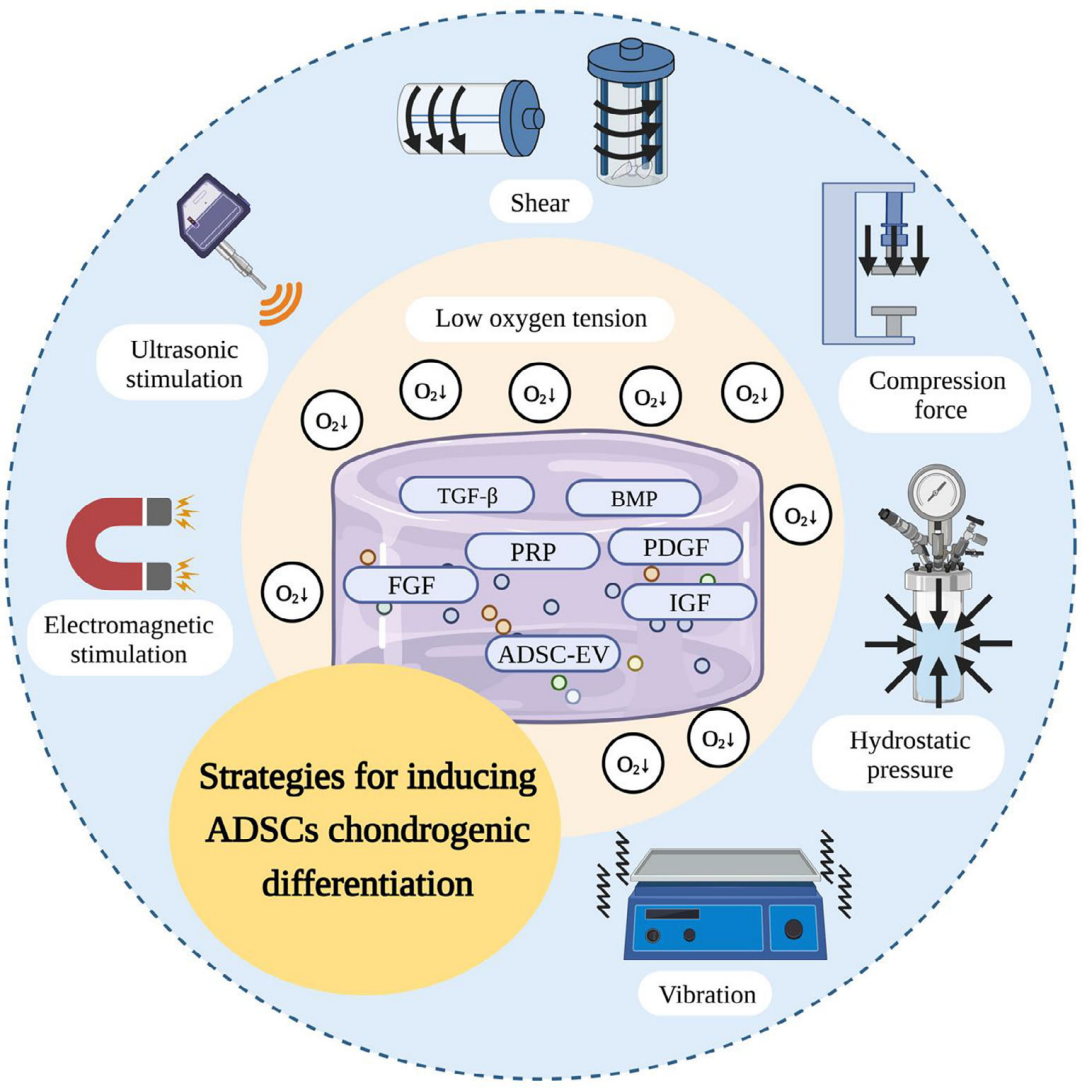
Strategies for inducing the chondrogenic differentiation of adipose‐derived stem cells (ADSCs). ADSC‐EV, ADSC‐derived extracellular vesicle; BMP, bone morphogenetic protein; FGF, fibroblast growth factor; IGF, insulin‐like growth factor; PDGF, platelet‐derived growth factor; PRP, platelet‐rich plasma; TGF‐β, transforming growth factor β.

For the construction of ideal highly bionic tissue‐engineered cartilage, it is not enough to consider the selection and design of seed cells and bio‐ink. It is also crucial to successfully induce seed cells such as ADSCs to differentiate into CCs and secrete cartilage ECM. Researchers have attempted to achieve optimal cartilage by simulating the natural physicochemical environment in vivo, such as adding appropriate growth factors, applying biomechanical stimuli, and reducing oxygen tension (Figure 6).

### 4.1 Biochemical additives for chondrogenic differentiation

#### 4.1.1 Growth factors and other biochemical additives

Growth factors play a crucial role in intercellular signalling, directing the behaviour and inducing the differentiation of MSCs. With the addition of various growth factors, the biomechanical properties of the constructed cartilage tissue, as well as the content of the matrix components, can be improved. Previous studies on cartilage tissue engineering have shown that growth factors, including TGF‐β, BMP, IGF, FGF, and platelet‐derived growth factor (PDGF), significantly improve the chondrogenic potential of MSCs.

It was found that TGF‐β3 could effectively stimulate the expression of ACAN, COL II, and SOX9 and favour the chondrogenesis of BMSCs,^87^ while the combination of BMP‐6 increased chondrogenic gene expressions on both BMSCs and ADSCs (with greater enhancement on ADSCs).^88^ Besides, a hybrid composite scaffold of SF and cartilage dECM with sequential delivery of TGF‐β3 could mimic the regenerative microenvironment to enhance the chondrogenic differentiation of ADSCs in vitro.^89^ In the study of Ma et al.,^90^ TGF‐β1 or/and BMP‐14 could induce BMSCs to express COL II, ACAN, and SOX9, with enhanced cell migration and inhibited apoptosis and alleviate the laryngeal cartilage defect in rabbits, and adding both at the same time is even more effective. BMP‐2, BMP‐4, and BMP‐6 could enhance the cartilage formation of MSCs in vitro, and BMP‐2 was the most effective.^91^ Durbec et al.^92^ reported that a cocktail of soluble factors (BIT), including BMP‐2, insulin, and triiodothyronine (T3), could induce the re‐differentiation of CCs and synthesize mature chondrogenic markers. In the study of Richmon et al.,^93^ FGF‐2 and TGF‐β1 both alone and in combination led to the greatest proliferation of human nasoseptal CCs, compared with BMP‐2 and IGF‐1, and all of the growth factors except FGF‐2 were effective in retaining the morphology of CCs. BMP‐6 could be a potent inducer of chondrogenesis in ADSCs, up‐regulating the expression of cartilage‐specific matrix components, namely COL II and ACAN, while inhibiting the expression of the hypertrophic CCs marker COL X, but at the same time, BMSCs exhibited increased expression of COL X and a hypertrophic phenotype.^94^, ^95^ Growth differentation factor (GDF)‐5, also called BMP‐14 or cartilage‐derived morphogenetic protein 1, could enhance the chondrogenesis of BMSCs,^96^ and the cartilage repair effect of BMSCs‐laden scaffold.^97^ Besides, in the study of Han et al.,^98^ GDF‐5 could also induce chondrogenic differentiation of ADSCs in vitro, especially at a concentration of 100 μg/mL. However, GDF‐5 also promotes osteogenesis and hypertrophy, limiting its therapeutic utility.^96^ To circumvent this problem, Mang et al. engineered M1673, a GDF‐5 mutant with lower hypertrophic and osteogenic properties, retaining the anabolic and anti‐catabolic effects of GDF‐5 on CCs at the same time.^99^ PDGF‐BB could promote the migration of BMSCs in fibrin‐HA and HA‐Tyramine hydrogels with different crosslinking densities in vitro.^31^ Tumour necrosis factor α (TNF‐α) exposure during MSCs expansion could modulate SOX11 levels and WNT/β‐catenin signalling, increasing the chondrogenic differentiation capacity.^100^

Besides adding growth factors directly, chondrogenic genes have been transfected into MSCs to increase growth factor secretion and promote chondrogenic differentiation. Liu et al.^101^ testified that ADSCs with BMP‐14 gene transfer by adenovirus could enhance chondrogenic differentiation of ADSCs and accelerate cartilage defect repair in rabbits. Raftery et al.^102^ reported that delivering the SOX‐Trio of genes (SOX‐5, SOX‐6, and SOX‐9) could effectively instruct MSCs to differentiate toward CCs without TGF‐β3 supplementation and even inhibit the activation of endochondral ossification program. The study of Xu et al.^103^ showed that overexpression of SOX11 in MSCs by lentivirus‐mediated gene transfer leads to accelerated cartilage defect healing in rats by regulating β‐catenin at the post‐transcriptional level through protein–protein interaction. Inducing CCN4 over‐expression using adenovirus encoding CCN4, Yoshioka et al.^104^ found that CCN4 has a positive influence on chondrogenic differentiation of BMSCs by modulating the effects of TGF‐β3, and adding CCN4 directly did not have any function for osteo‐chondrogenesis. In the study of Chen et al.,^105^ adenovirus‐mediated gene transfection of bFGF transfection significantly promoted CCs proliferation, increasing the synthesis of GAG and COL II, and multiple‐gene transfection in different combinations (bFGF and interleukin [IL]‐1 receptor antagonist protein or IGF‐1) showed better results than bFGF transfection alone.

In addition, there are also other attractive biochemical factors beneficial to MSCs differentiation and cartilage regeneration (Table 4).

**TABLE 4:** Biochemical factors beneficial to MSCs differentiation and cartilage regeneration.

Biochemical factor	Mechanics and effects	Reference
Matrilin‐3 (ECM component in cartilage tissue)	Increased chondrogenic differentiation of ADSCs, enhanced the cartilage regeneration at osteochondral defect sites (without cytotoxicity and altering proliferation)	^106^
L‐ascorbic acid	Promoted cell adhesion and cell proliferation, entered the cell through sodium‐dependent vitamin C transporter and activated COL2A1 , promoting chondrogenic differentiation of ADSCs	^107^, ^108^
Strontium ranelate (antiosteoporosis drug)	Promoted BMSCs chondrogenic differentiation by inhibiting the WNT/β‐catenin signalling	^109^
Simvastatin (clinically used lipid‐lowering agent)	Promoted chondrogenic differentiation of ADSCs by up‐regulating BMP‐2	^110^, ^111^
Icaritin	Induced the chondrogenic differentiation of BMSCs in rats (in combination with GDF‐5), promoted the chondrogenic differentiation of BMSCs in vitro	^112^
Y27632 (inhibitor for Rho‐associated coiled‐coil containing protein kinase)	Increased the differentiation of chondroprogenitors, prevented the expression of hypertrophic marker of MSCs in 3D printed PU/HA scaffolds	^113^
NSC23766 (Rac1 inhibitor)	Facilitated the efficiency of TGF‐β3‐induced ADSCs chondrogenesis by suppressing CCs from undergoing hypertrophy and calcification	^114^

Abbreviations: ADSCs, adipose‐derived stem cells; BMP‐2, bone morphogenetic protein 2; BMSCs, bone marrow‐derived mesenchymal stem cells; CCs, chondrocytes; ECM, extracellular matrix; GDF‐5, growth differentiation factor 5; HA, hyaluronic acid; MSCs, mesenchymal stem cells; PU, polyurethane; TGF‐β3, transforming growth factor β3.

#### 4.1.2 Natural combinations of biofertilizers

In addition to adding a variety of growth factors, the growth and chondrogenic differentiation of ADSCs can also be promoted by directly adding “natural combinations of growth factors” such as platelet‐rich plasma (PRP) and ADSC‐derived EVs (ADSC‐EVs).

PRP is a part of whole blood with a high concentration of platelets. In addition to the platelets, PRP also contains a large number of cytokines, such as TGF‐β, IGF‐1, epidermal growth factor (EGF), PDGF, and so forth.^115^, ^116^ As described in the previous part, these cytokines play an important role in supporting the growth and differentiation of MSCs.^117^ Several investigations have revealed that PRP could maintain the chondrogenic differentiation capacity of MSCs, promote cell migration and proliferation and maintain an immunosuppressive state.^118^ PRP promoted cell migration of BMSCs via stimulating the signalling pathway of PI3K‐AKT.^118^ And, compared with common chondrogenic induction agents (TGF‐β1, dexamethasone, and vitamin C), autologous PRP could be more effective in promoting the chondrogenesis of BMSCs.^115^ Combined with hydrogels, PRP has been widely used for cartilage tissue engineering. PRP can be used as an autologous source of growth factors, and the addition of PRP to SF‐based bio‐ink compensates for the lack of SF bioactivity.^119^ PRP induction medium on the SF scaffold could effectively promote cell adhesion and cell proliferation and increase the concentration of collagen and glycosaminoglycan and promote the chondrogenic differentiation of ADSCs in vitro.^108^, ^119^, ^120^ In addition to SF, the selection of GelMA as the polymeric component allows for long‐term, constant rate growth factor release and bioactive protection of PRP with high mechanical properties in the study of Irmak et al.^121^ Besides, in the study of Jiang et al.,^122^ PRP‐GelMA hydrogels participated in immune regulation and M1‐to‐M2 transition of macrophages chemotaxis of BMSCs, coordinating dynamic immune regulation and promoting the migration and osteogenic and chondrogenic differentiation of BMSCs.

EVs are cell‐secreted packages that deliver cargo to target cells to effect functional and phenotypic changes.^123^ They are secreted by many different types of cells, including ADSCs, and are generically divided into microvesicles (50–1000 nm) and exosomes (30–100 nm).^123^, ^124^ Some studies have shown that ADSC‐EVs can enhance chondrogenesis and chondrogenic differentiation of seed cells. In the study of Li et al.,^125^ the ADSC‐EVs stimulate the migration, proliferation, and chondrogenic and osteogenic differentiation of BMSCs in vitro as well as cartilage and bone regeneration in a mouse model more than the BMSC‐EVs or SMSC‐EVs. The tissue origin may contribute to the distinct protein profiles among the three types of EVs, which induced cartilage and bone regenerative capacities by potential mechanisms of regulating signalling pathways, including focal adhesion, ECM‐receptor interaction, actin cytoskeleton, cAMP, and PI3K‐AKT signalling pathways.^125^ However, apparently few differences were found between ADSC‐EVs and BMSC‐EVs in the study of Gorgun et al.,^126^ and both of them could possess immunomodulatory, pro‐regenerative, pro‐angiogenic, and anti‐apoptotic properties. In the study of Xue et al.,^127^ the hypoxic ADSCs‐derived EVs improved the proliferation and chondrogenic differentiation of CPCs for cartilage tissue engineering. These results indicate that the ADSCs not only play a role in cartilage tissue engineering as seed cells but also serve as the source of exosomes, a regulator promoting cartilage regeneration. Disappointingly, in the study of Oh et al.,^128^ ADSCs significantly improved auricular cartilage regeneration, while their secretome could not be retained in the long term in the open, surgically created, auricular cartilage defect and work. When compared with bone regeneration, the research of ADSC‐EVs in cartilage regeneration is still somewhat under‐researched, especially when ADSCs are used as seed cells, which needs further research.

### 4.2 Physical stimulation

Physical stimulation and the application of bioreactor play an important role in cartilage tissue engineering. Appropriate physical stimulation helps the constructed tissue acquire histological and biomechanical properties close to natural cartilage tissue. There is a lack of studies on physical stimulation promoting ADSCs chondrogenesis at present, while many studies focus on BMSCs for reference.

Depending on the mode and direction of mechanical stimulation, it can be roughly classified into three types: shear, compression force, and hydrostatic pressure (HP).^129^ In the study of Shahmoradi et al.,^130^ applying HP on BMSCs seeded in demineralized bone matrix scaffolds could decrease differentiation to fibroblasts and osteogenic differentiation, and induce chondrogenic differentiation. Kang et al. combined ADSCs with articular cartilage dECM, accelerating the proliferation and differentiation of ADSCs by taking advantage of the shear stress generated by fluid convection with a rotating bioreactor.^131^ Zhu et al. demonstrated that a dynamic rotating wall vessel bioreactor system constructing relatively low shear stress could accelerate the mass transfer rate of glucose and TGF‐β2 around the cell‐hydrogel constructs, improving the proliferation and differentiation of ADSCs and significantly shortening the time for cell construct organization.^132^ Besides, additional compressive force during dynamic culture can improve the chondrogenic differentiation of human BMSCs. However, Guo et al.^133^ reported that static culture was preferred for the chondrogenic differentiation of human BMSCs compared with dynamic culture with shear stress only. In the physiological state, the nasal cartilage is subjected to intrinsic or extrinsic compression forces daily. In the study of Lin et al.,^134^ dynamic compressive loading could promote the cartilage formation of BMSCs encapsulated in the HAMA scaffold and enhance cartilage healing of the osteochondral defect. Intermittent mechanical stimulation (unload phases of 10 s each 180 cycles) provided by a compression bioreactor promotes the migration of BMSCs on the scaffold, whereas continuous mechanical stimulation decreases cell viability.^135^ Furthermore, the application of perfusion and dynamic compression (0.5 Hz, 2 h action +4 h pause/cycle, 4 cycle/day) could benefit the chondrogenic differentiation of BMSCs and achieve significantly higher compressive modulus.^136^

In addition to bioreactors that provide the usual mechanical stimulation described above, low amplitude high frequency vibration loading, as a model of mechanical stimulation, has been demonstrated to promote chondrogenic differentiation of bone marrow stem cells with the involvement of the β‐catenin signalling pathway.^137^ Besides, electromagnetic field (EMF) and ultrasonic stimulation are also used in promoting the chondrogenesis of MSCs. The EMF can be divided into static and pulsed magnetic fields according to the changing pattern of the magnetic field over time.^129^ Appropriate electromagnetic stimulation can promote chondrogenic differentiation of MSC and enhance cartilage repairs, such as pulsed low‐frequency EMF^138^ and moderate‐intensity SMF (0.6 T^139^ or 40 mT^140^). In the absence of exogenous addition of TGF‐β, continuous low‐intensity ultrasound promotes cartilage differentiation in MSCs by up‐regulating the expression of the transcription factor SOX9, depending on the phosphorylation of ERK1/2.^141^

### 4.3 Hypoxic environment

The average partial pressure of oxygen measured at the septum and inferior turbinate was 10.5 ± 10.1 mmHg (1.4% ± 1.3%) and 27.6 ± 12.4 mmHg (3.6% ± 1.6%), respectively.^142^ At the same time, the physiological oxygen level within human articular cartilage ranges from 2% to 5% and 7% in the bone marrow.^143^ Most traditional tissue culture incubators operate at atmospheric oxygen levels (20%) that are actually non‐physiological and hyperoxygen environments for seed cells.

In the study of Scotti et al.,^144^ human articular CCs displayed a positive response to low oxygen culture, while human nasal CCs were only slightly affected by oxygen percentage. However, it has a significant influence on MSCs. In particular, increasing evidence has demonstrated that low oxygen tension promotes cell proliferation,^76^ enhances chondrogenic differentiation capacity,^145^ and increases migration ability^146^ but inhibits osteogenic differentiation.^147^ The study of Shearier et al.^148^ showed that the biochemical components of cartilage in vitro are more similar to natural tissue when cultured under hypoxic conditions. Co‐culture with ADSCs and CCs under hypoxia was shown to induce or enhance ADSCs to chondrogenic differentiation successfully. Specifically, chondrogenic marker genes: ACAN, COL II, and SOX9 were remarkably enhanced to express in both ADSCs and CCs after crosstalk under low oxygen tension.^76^ By increasing the level of hypoxia‐inducible factor 1α (HIF‐1α), vascular endothelial growth factor A/B (VEGF‐A/B), BMP‐2/‐4/‐6, FGF‐2, and IGF‐1, hypoxia pretreatment could increase the proliferation and chondrogenic differentiation of ADSCs but decrease the osteogenic differentiation of ADSCs.^76^, ^146^ Besides, Yasui et al.^145^ found that hypoxic treatment (5% oxygen) of human SMSCs prevented senescence induction, evidenced by 2.70‐fold‐lower expression levels for P16.

## 5 SUMMARY AND FUTURE OUTLOOKS

Three‐dimensional bioprinting technology is developing rapidly, and different improved printing systems and construction methods are emerging. Zhou et al. developed a multi‐axis robot‐based bioprinting system supporting natural cell function preservation and cardiac tissue fabrication, enabling cell printing on 3D complex‐shaped vascular scaffolds from all directions.^149^ Lan et al.^150^ reported an in vitro nasal cartilage tissue engineering method via the freeform reversible embedding of suspended hydrogel 3D bioprinting. Hwang et al.^151^ presented a 3D bioprinting platform on a high‐throughput scale, which is capable of rapid, continuous 3D printing of constructs with small sizes (<10 μ) and complex shapes and controlling the mechanical property. Although some of them are not yet used for cartilage tissue engineering, they provide more ideas and are expected to be used in the future to print more complex and nuanced cartilage tissue or complex systems containing cartilage tissue efficiently.

Different hydrogel materials for 3D bioprinting have emerged in recent years, and different combinations of bio‐ink have been explored. Current studies in the field mainly focus on mixtures of bio‐ink with “good biocompatibility” (such as dECM, HA) and “photocrosslinking properties” (such as HAMA, GelMA, PEGDMA) or “good mechanical properties “(such as silk protein, synthetic materials). However, no ink formulation has been proven to have absolute dominance. What is certain is that the main trend is to construct tissue‐engineered cartilage that is more histologically bionic, which can be achieved with the help of layered design and switching printing modes (bionic bio‐ink printing paths and fibre arrangement). Moreover, ADSCs have become one of the most popular seed cells in cartilage 3D bioprinting, owing to their abundance, excellent proliferative potential, minimal morbidity during harvest, and lack of ethical considerations limitations. In addition, the co‐culture of ADSCs and CCs is also commonly used to achieve better chondrogenesis. At the same time, the optimal selection of the chondroblast differentiation mode of the printed body has not been determined. It is believed that with the efforts of researchers, a standardized protocol for construct handling and implantation will be produced in the future.

## CONFLICT OF INTEREST STATEMENT

The authors declare no conflict of interest.

## Data Availability

Data sharing is not applicable to this article as no new data were created or analyzed in this study.
